# Supplementary material for: Forcing the reversibility of a mechanochemical reaction
Source: Nat Commun. 2018 Aug 8;9:3155. doi: 10.1038/s41467-018-05115-6 (PMC6082871; doi:10.1038/s41467-018-05115-6)
Supplement: Supplementary file 1 — Supplementary Information [file 41467_2018_5115_MOESM1_ESM.pdf]

Supplementary Information for:

Forcing the reversibility of a mechanochemical reaction

Amy E. M. Beedle[1*], Marc Mora[1*], Colin T. Davis[2], Ambrosius P. Snijders[2], Guillaume Stirnemann[3] and Sergi Garcia-Manyes[1, 4]

[1]Department of Physics and Randall Centre for Cell and Molecular Biophysics, King's College London, WC2R 2LS, London, UK.
[2]The Francis Crick Institute, Protein analysis and Proteomics Science Technology Platform, 1 Midland Road, London NW1 1AT, UK
[3] CNRS Laboratoire de Biochimie Théorique, Institut de Biologie Physico-Chimique, Univ. Paris Denis Diderot, Sorbonne Paris Cité, PSL Research University, 13 rue Pierre et Marie Curie, 75005, Paris, France
[4]The Francis Crick Institute, 1 Midland Road, London NW1 1AT, UK.

* These authors contributed equally to the work
Correspondence: S.G.-M. (sergi.garcia-manyes@kcl.ac.uk)

Supplementary Figures

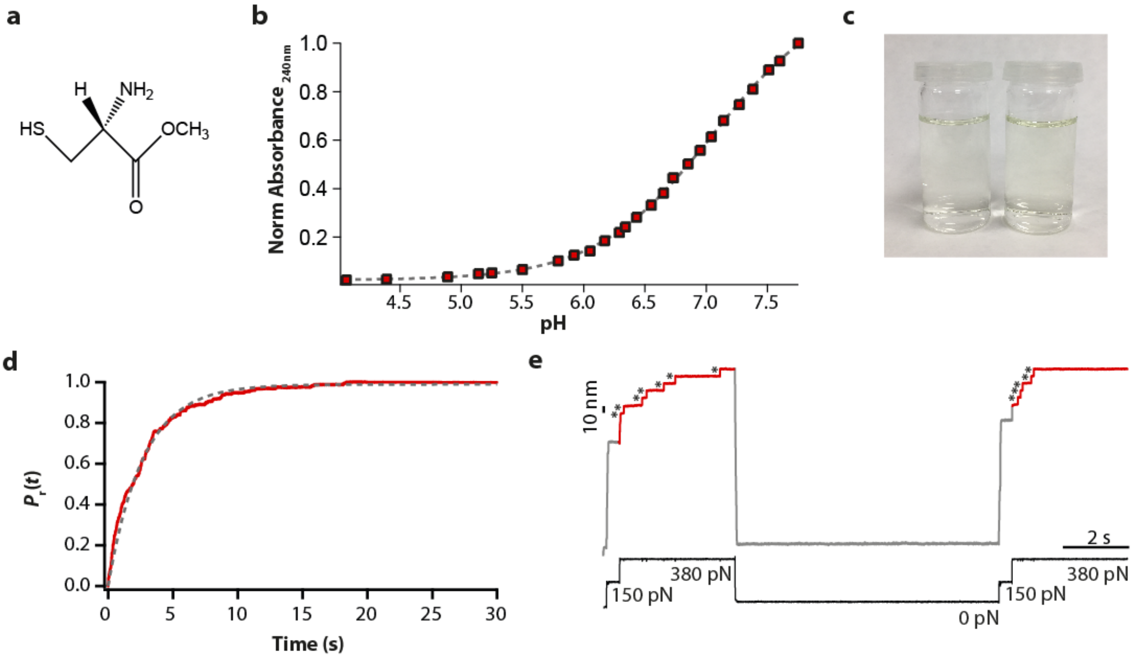

Supplementary Figure 1. Cysteine methyl ester, Cys-ME. (a) Chemical structure of cysteine-methyl-ester. **(b)** Absorbance measurements at λ=240nm as a function of pH reveal a pK_a = 7.0 **(c)** Qualitative comparison between the Ellman's blank (*left*) and the Ellman's with [S^-] = 0.25 µM of Cys-ME (*right*). **(d)** The rate of disulfide reduction at 350 pN at [S^-] = 2.16 mM is *r* = 0.40 ± 0.05 s^{-1} (*n*=24). **(e)** Disulfide bond reduction by cysteine-methyl-ester yields a high percentage of reformation of 43.6 ± 5.0 % (n=16). All errors: s.d.

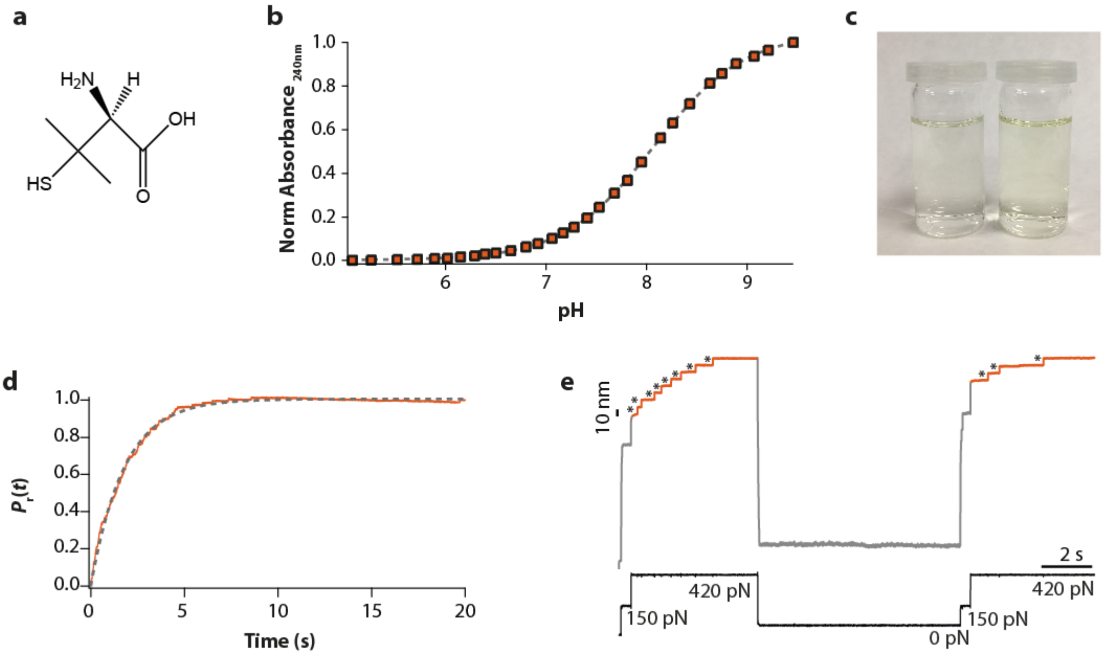

Supplementary Figure 2. Penicillamine. (a) Chemical structure of penicillamine. **(b)** Absorbance measurements at λ=240nm as a function of pH reveal a pK_a = 8.0 **(c)** Qualitative comparison between the Ellman's blank (*left*) and the Ellman's with [S^-] = 0.25 µM of penicillamine (*right*). **(d)** The rate of disulfide reduction at 350 pN at [S^-] = 2.39 mM is r = 0.58 ± 0.06 s^{-1} (n=13). **(e)** Disulfide bond reduction by penicillamine yields a reformation percentage of 33.9 ± 2.9 % (n=35). All errors: s.d.

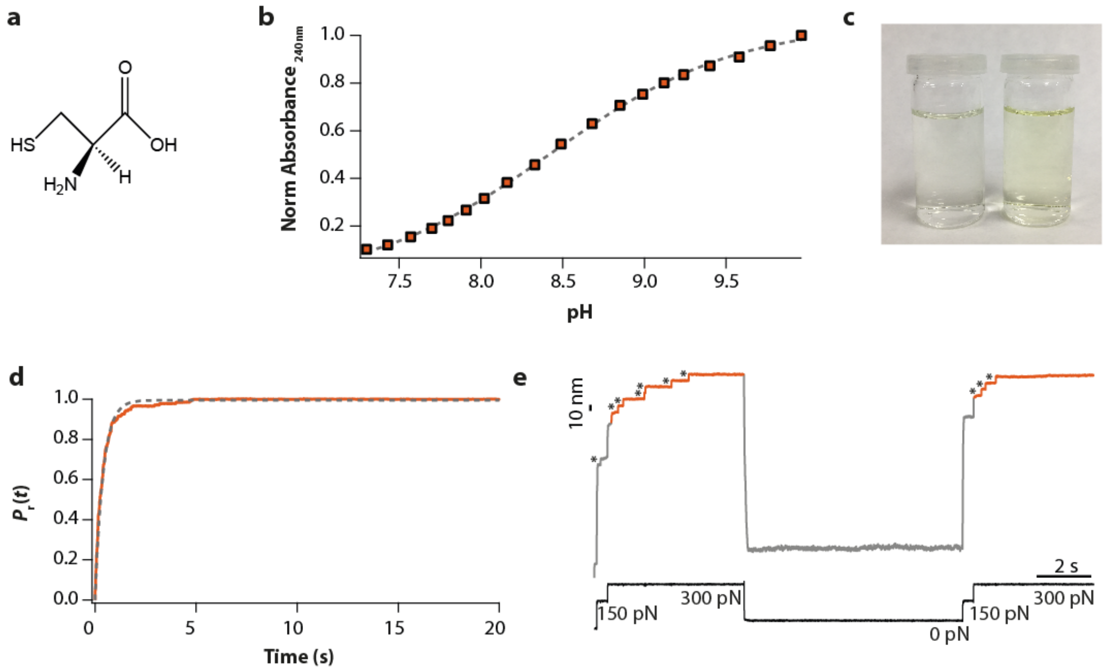

Supplementary Figure 3. L-cysteine. (a) Chemical structure of l-cysteine. **(b)** Absorbance measurements at λ=240nm as a function of pH reveal a pK_a = 8.4 **(c)** Qualitative comparison between the Ellman's blank (*left*) and the Ellman's with [S^-] = 0.25 µM of cysteine (*right*). **(d)** The rate of disulfide reduction at 350 pN at [S^-] = 2.0 mM is r = 2.48 ± 0.22 s^{-1} (n=23). **(e)** Disulfide bond reduction by l-cysteine yields a reformation percentage of 36.7 ± 2.9 % (n=30). All errors: s.d.

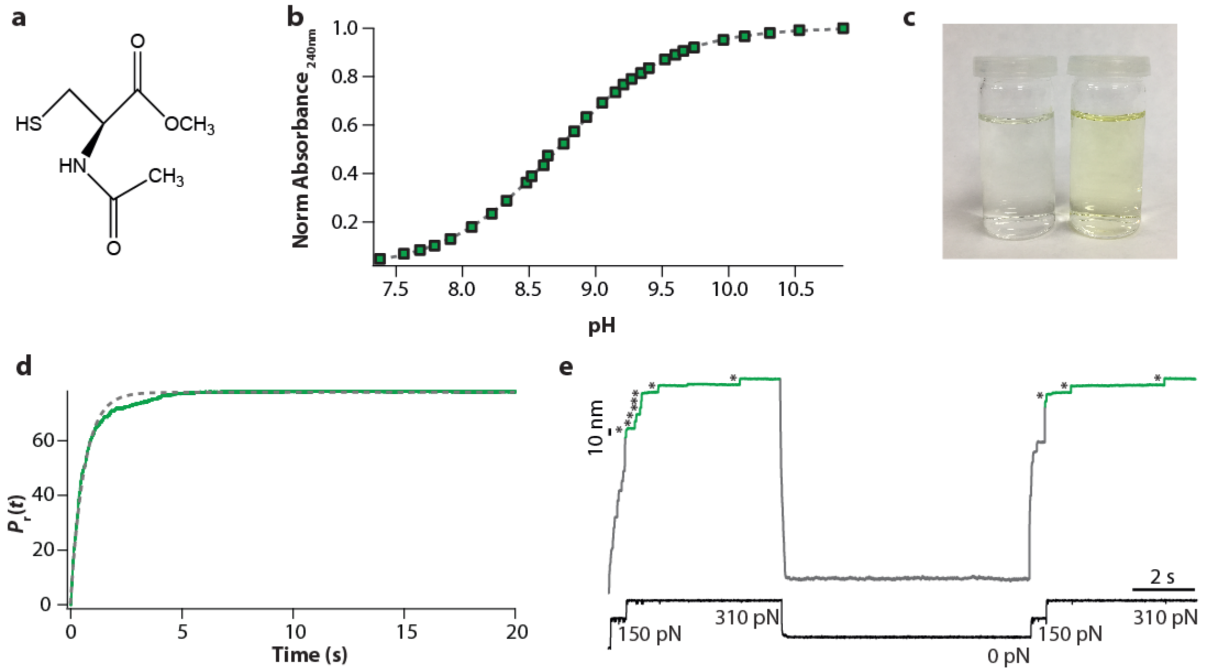

Supplementary Figure 4. NAC-methyl-ester. (a) Chemical structure of n-acetyl-cysteine-methyl-ester. **(b)** Absorbance measurements at λ=240nm as a function of pH reveal a pK_a = 8.7 **(c)** Qualitative comparison between the Ellman's blank (*left*) and the Ellman's with [S^-] = 0.25 µM of NAC-methyl-ester (*right*). **(d)** The rate of disulfide reduction at 350 pN at [S^-] = 0.48 mM is r = 1.69 ± 0.21 s^{-1} (n=34). **(e)** Disulfide bond reduction by NAC-methyl-ester yields a reformation percentage of 17.9 ± 3.1 % (n=24). All errors: s.d.

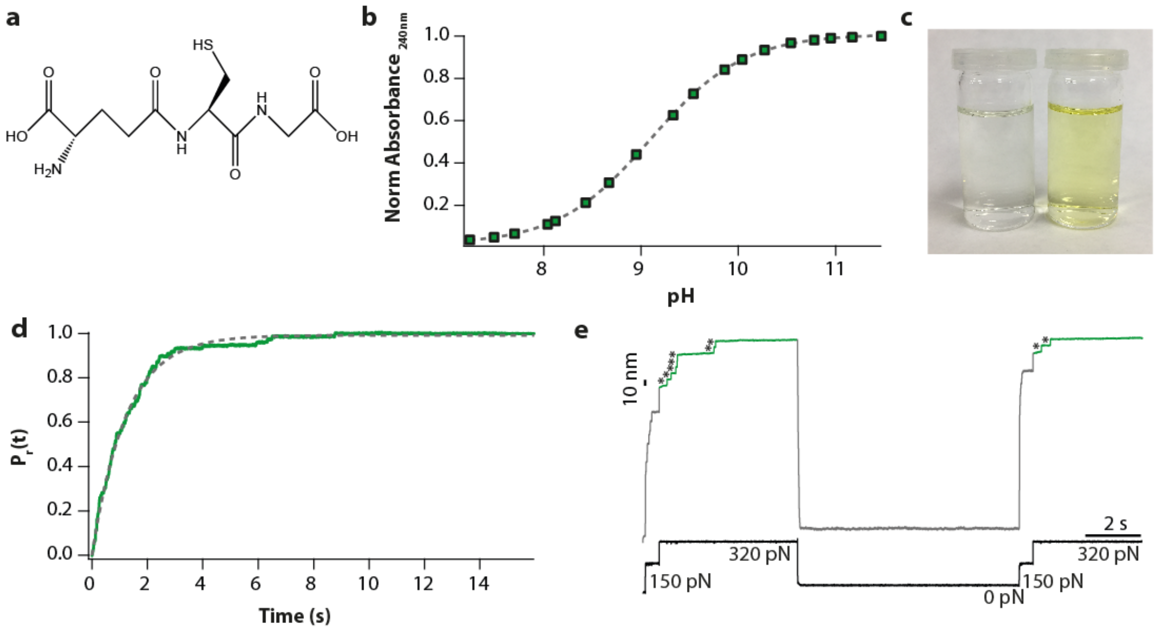

Supplementary Figure 5. Glutathione. (a) Chemical structure of glutathione. **(b)** Absorbance measurements at λ=240nm as a function of pH reveal a pK_a = 9.1 **(c)** Qualitative comparison between the Ellman's blank (*left*) and the Ellman's with [S^-] = 0.25 µM of glutathione (*right*). **(d)** The rate of disulfide reduction at 350 pN at [S^-] = 0.49 mM is r = 0.83 ± 0.07 s^{-1} (n=17). **(e)** Disulfide reduction by glutathione yields a reformation percentage of 16.8 ± 2.9 % (n=32). All errors: s.d.

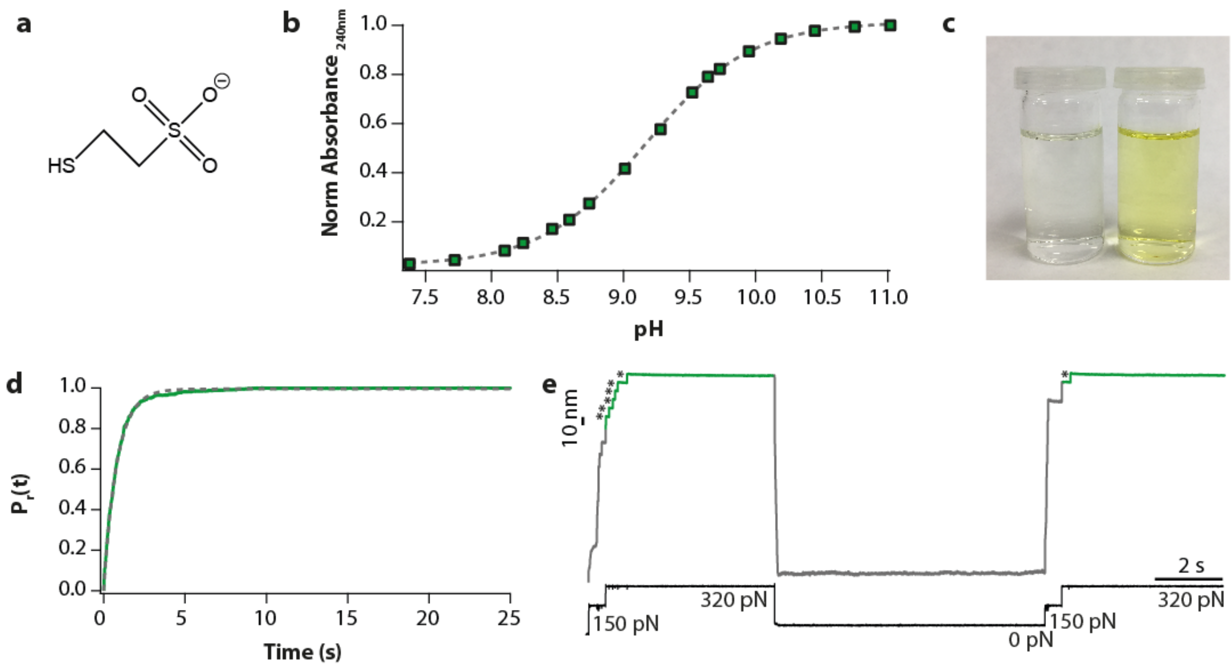

Supplementary Figure 6. Mesna. (a) Chemical structure of Mesna. **(b)** Absorbance measurements at λ=240 nm as a function of pH reveal a pK_a = 9.2 **(c)** Qualitative comparison between the Ellman's blank (*left*) and the Ellman's with $[S^-]$ = 0.25 µM of Mesna (*right*). **(d)** The rate of disulfide reduction at 350 pN at $[S^-]$ = 0.5 mM is r = 1.37 ± 0.10 s^{-1} (*n*=35). **(e)** Disulfide bond reduction by mesna yields a reformation percentage of 4.7 ± 2.0 % (*n*=33). All errors: s.d.

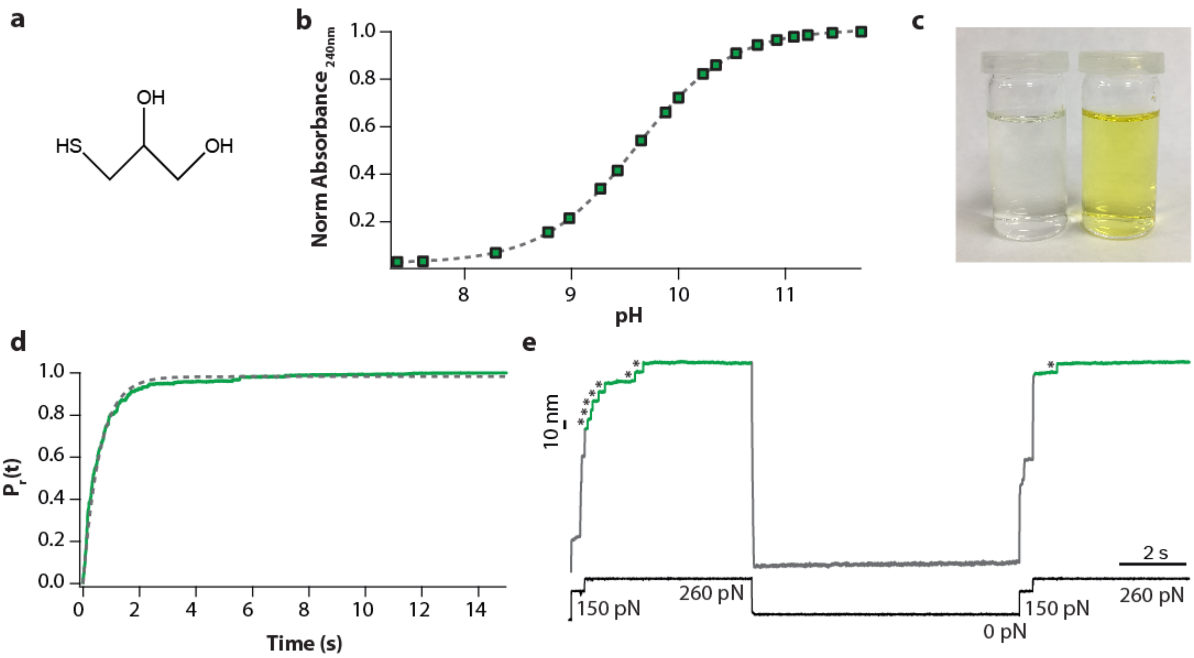

Supplementary Figure 7. Thioglycerol. (a) Chemical structure of 1-thiogylcerol. **(b)** Absorbance measurements at λ=240nm as a function of pH reveal a pK_a = 9.6 **(c)** Qualitative comparison between the Ellman's blank (*left*) and the Ellman's with [S^-] = 0.25 µM of thioglycerol (*right*). **(d)** The rate of disulfide reduction at 350 pN at [S^-] = 0.6 mM is r = 1.75 ± 0.16 s^{-1} (n=23). **(e)** Disulfide bond reduction by thioglycerol yields a reformation percentage of 6.5 ± 1.4 % (n=53). All errors: s.d.

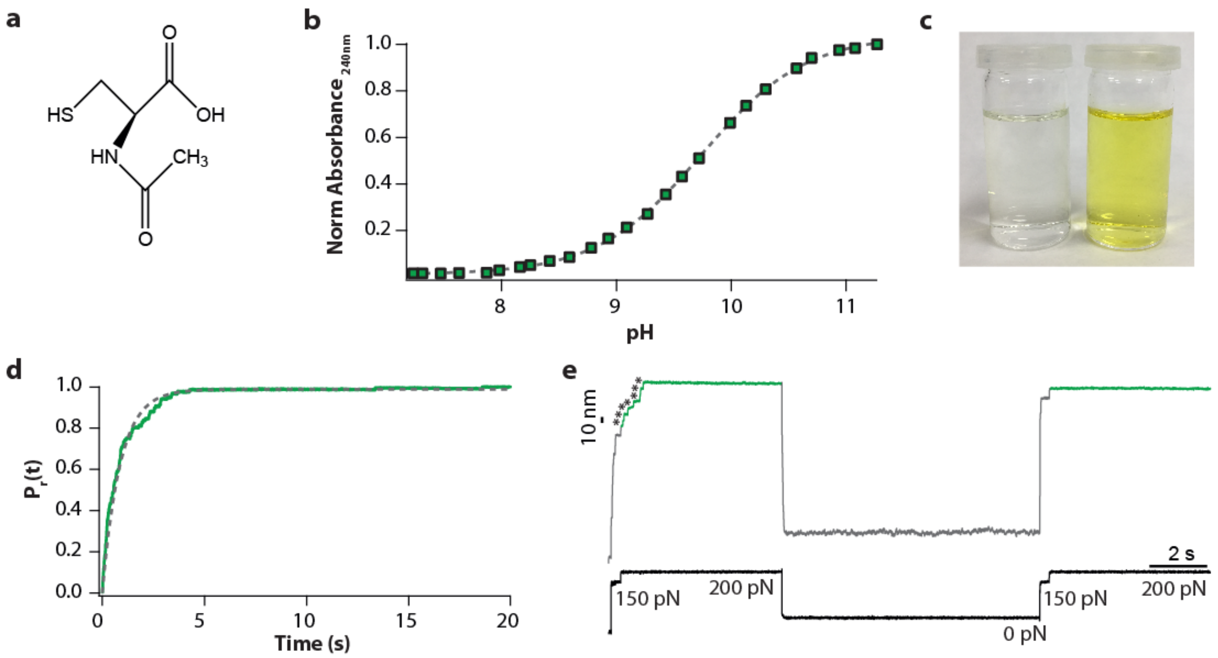

Supplementary Figure 8. NAC. (a) Chemical structure of NAC. **(b)** Absorbance measurements at λ=240nm as a function of pH reveal a pK_a = 9.7 **(c)** Qualitative comparison between the Ellman's blank (*left*) and the Ellman's with [S^-] = 0.25 µM of NAC (*right*). **(d)** Rate of disulfide reduction at 350 pN at [s-] = 0.2 mM is r = 1.16 ± 0.17 s^{-1} (n=17). **(e)** Disulfide bond reduction by NAC yields a reformation percentage of 8.6 ± 2.0 % (n=31). All errors: s.d.

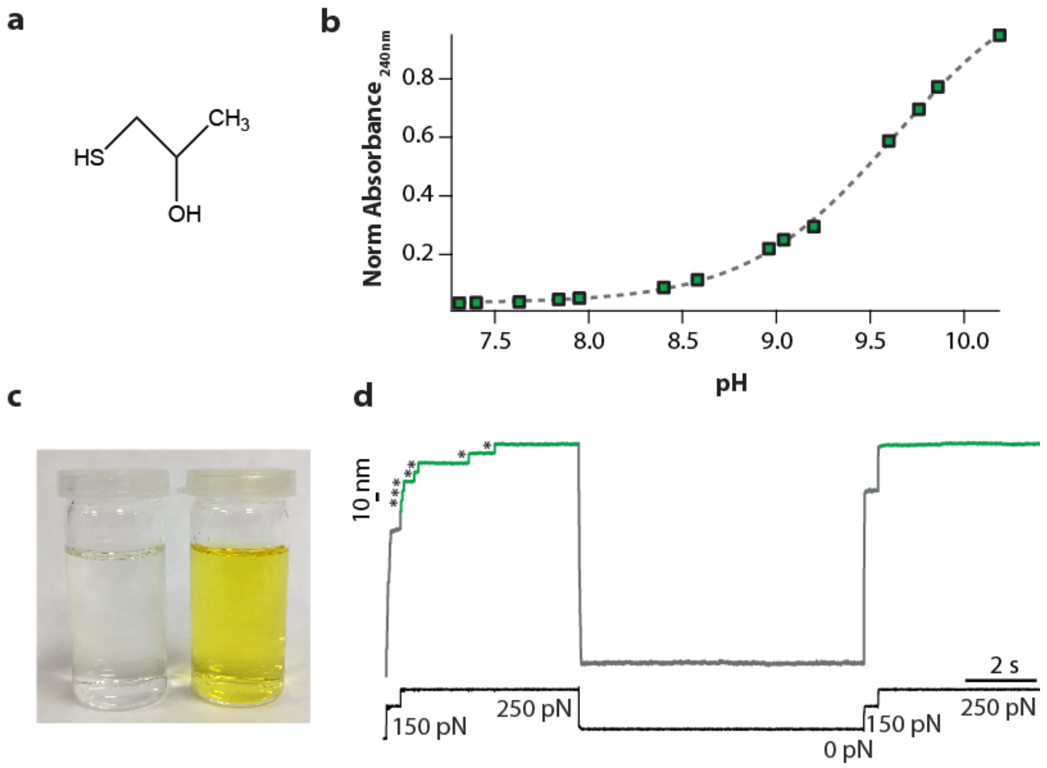

Supplementary Figure 9. 1-mercapto-2-propanol. (a) Chemical structure of 1-mercapto-2-propanol. **(b)** Absorbance measurements at λ=240nm as a function of pH reveal a pK_a = 9.8 **(c)** Qualitative comparison between the Ellman's blank (*left*) and the Ellman's with [S^-] = 0.25 μM of 1mercapto-2propanol (*right*). **(d)** Disulfide bond reduction by 1-mercapto-2-propanol yields a vanishingly small reformation percentage of 1.6 ± 0.9 % (*n*=39). Errors: s.d.

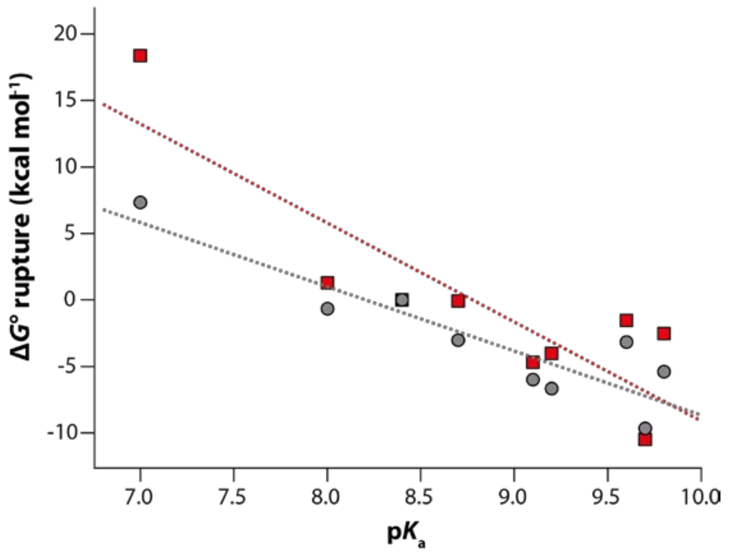

Supplementary Figure 10. The DFT calculated Δ*G*° values correlate well with the experimental pK_a determination. The inclusion of one explicit water molecule in the M06-2X functional leads to a good agreement between calculated and experimental pK_a value (grey circles, r^2 = 0.812). The B3LYP functional gives qualitatively similar results (red squares, r^2 = 0.755).

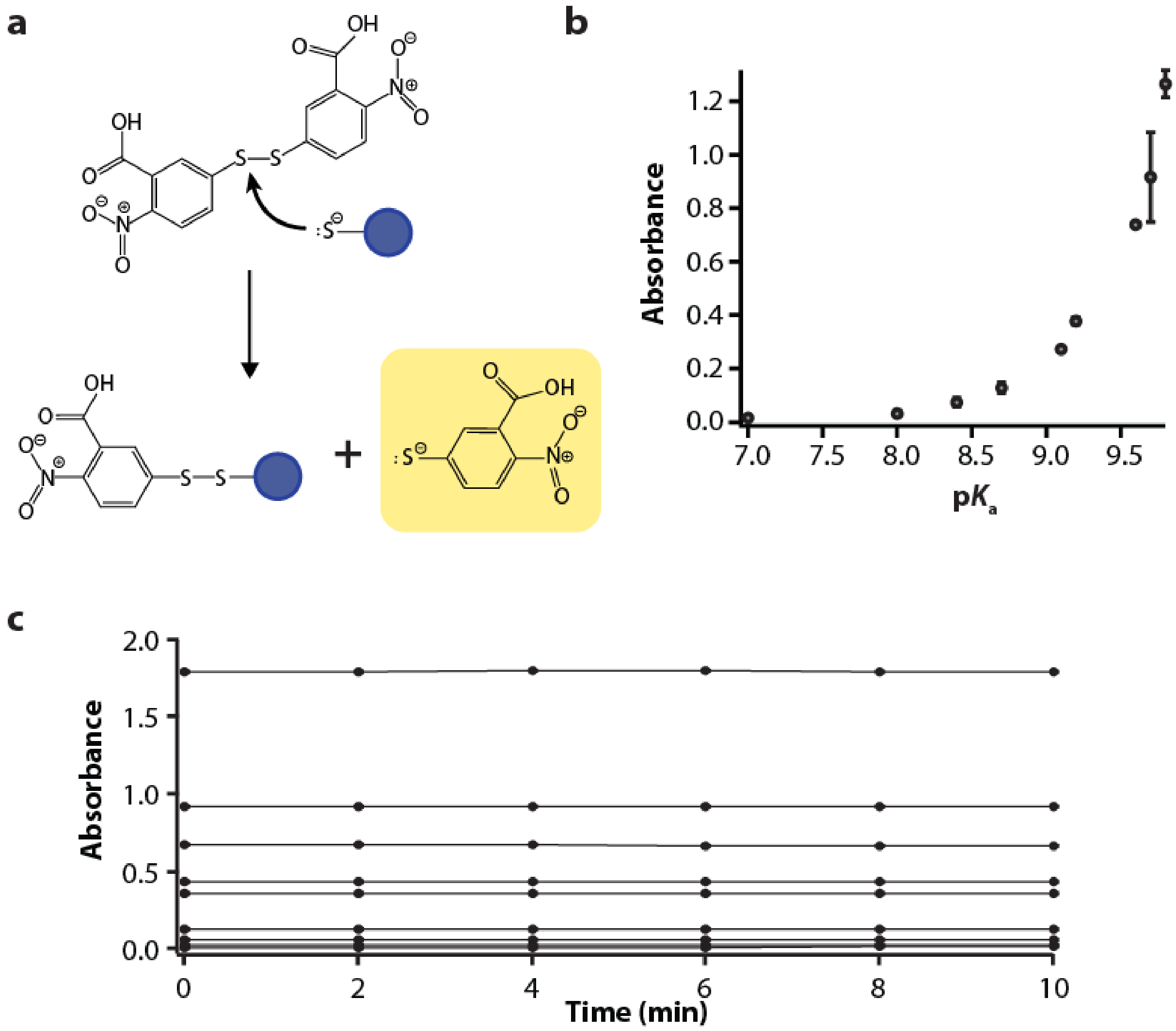

Supplementary Figure 11. Ellman's assay. (a) The cleavage of the disulfide bond in DTNB by a thiolate, gives rise to TNB, which is yellow. **(b)** The intensity of absorbance at λ = 412 nm for each compound scales with the pK_a of the thiol (n=3 for each compound; error bars: s.d). **(c)** The absorbance at λ = 412 nm for the Ellman's assay with each sulfur compound is invariant with time over the first 10 min. This indicates that the assay is under thermodynamic control.

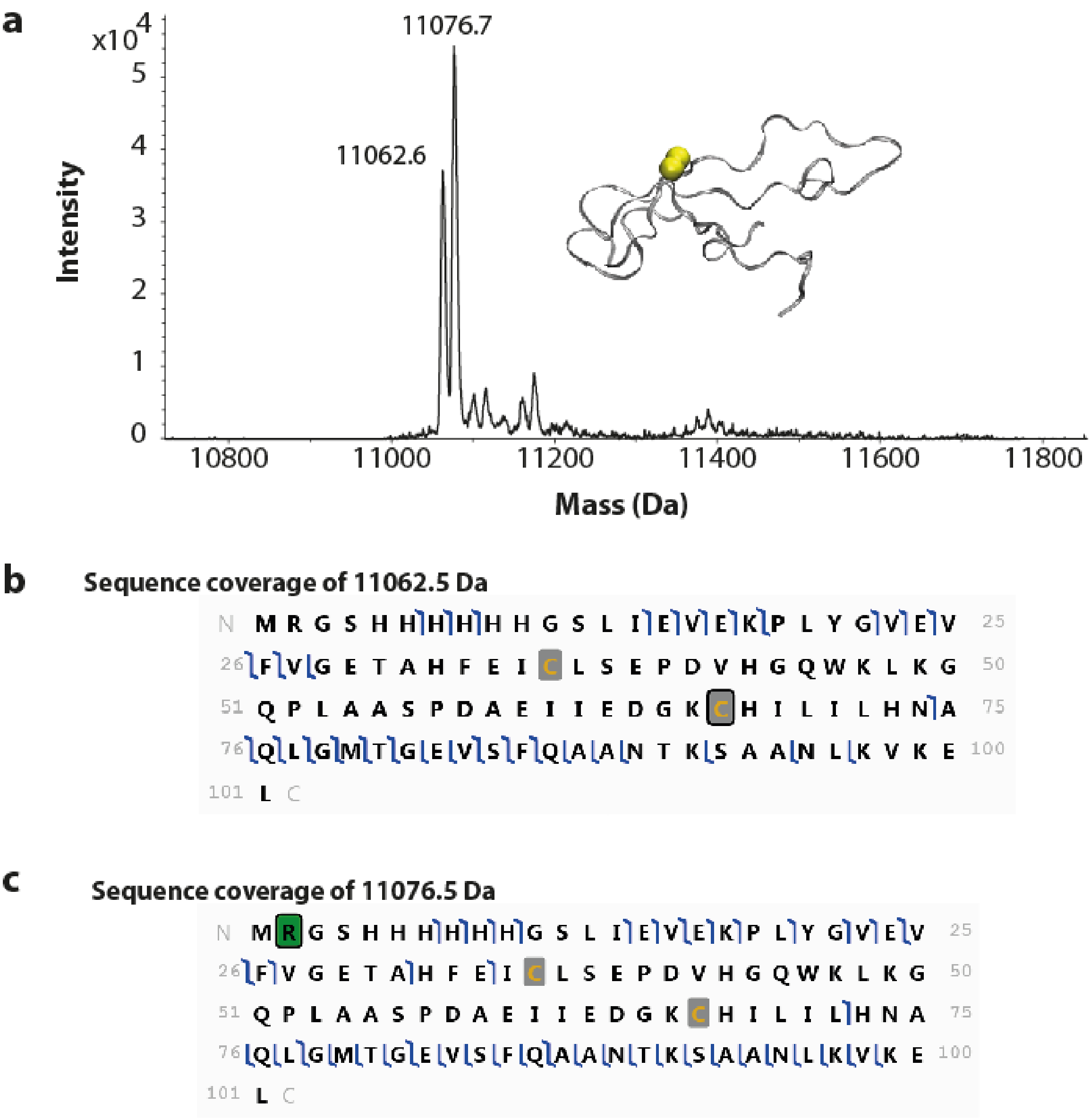

Supplementary Figure 12. Mass spectrometry characterization of I27$_{24/55}$. (a) The deconvoluted mass spectrum of intact I27$_{24/55}$ reveals a clear peak at a mass of ~11063 Da which corresponds to the whole oxidized protein. The peak at 11076 Da corresponds to the methylation of the protein. **(b)** Top down MS/MS experiments reveal that the methylation occurs within the purification tag. Collision Induced Dissociation (CID) of the 11062.5 Da peak identifies fragment ions corresponding to the I27$_{E24C\text{-}k55C}$ construct thereby confirming the sequence. See supplementary Table 4 for fragmentation peak values. **(c)** CID of +14 Da peak at 11076.5 Da also identifies fragment ion series corresponding to the I27$_{E24C\text{-}k55C}$ construct. b-ions corresponding to the N-terminal region of the sequence localise the +14 Da mass addition to the His-tag region (the protein purification tag). Blue separators indicate identified b- and y-ions in the fragmentation spectra. The oxidised cysteine residues are highlighted in grey and the possible methylation event (+14 Da) is indicated in green. See supplementary Table 5 for fragmentation peak values.

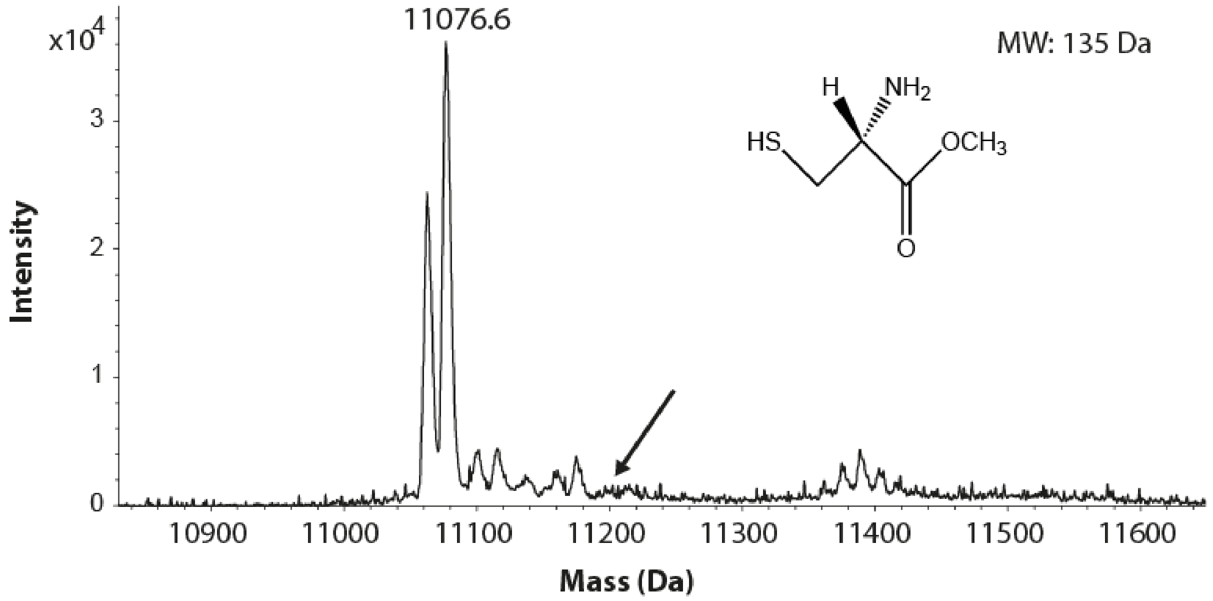

Supplementary Figure 13. Deconvoluted mass spectrum of I27$_{24/55}$ in the presence of cysteine-methyl-ester. The deconvoluted mass spectrum of I27$_{24/55}$ in the presence of cysteine-methyl-ester does not display any discernible peak corresponding to the modification of the protein. The black arrow indicates the anticipated location of the modification at a mass of 11212 Da.

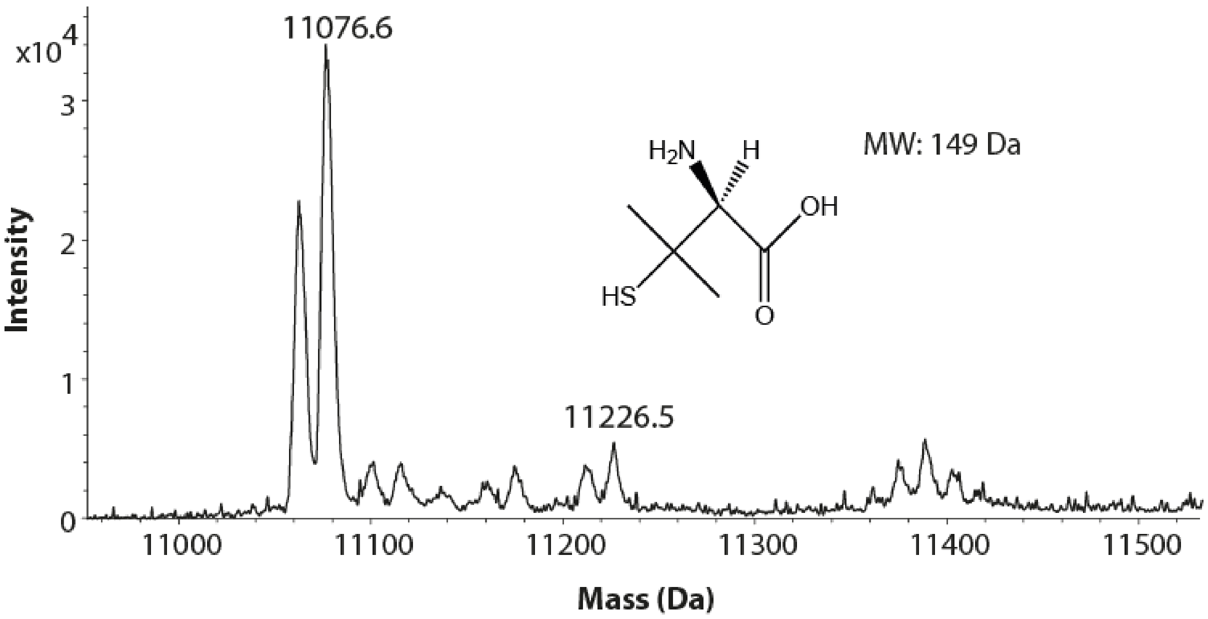

Supplementary Figure 14. Deconvoluted mass spectrum of I27$_{24/55}$ with the post-translational modification by penicillamine. The deconvoluted mass spectrum of I27$_{24/55}$ in the presence of penicillamine displays two peaks at a mass of 11212 Da and 11226 Da, corresponding to the modification of both the non-methylated and methylated protein at a single cysteine residue.

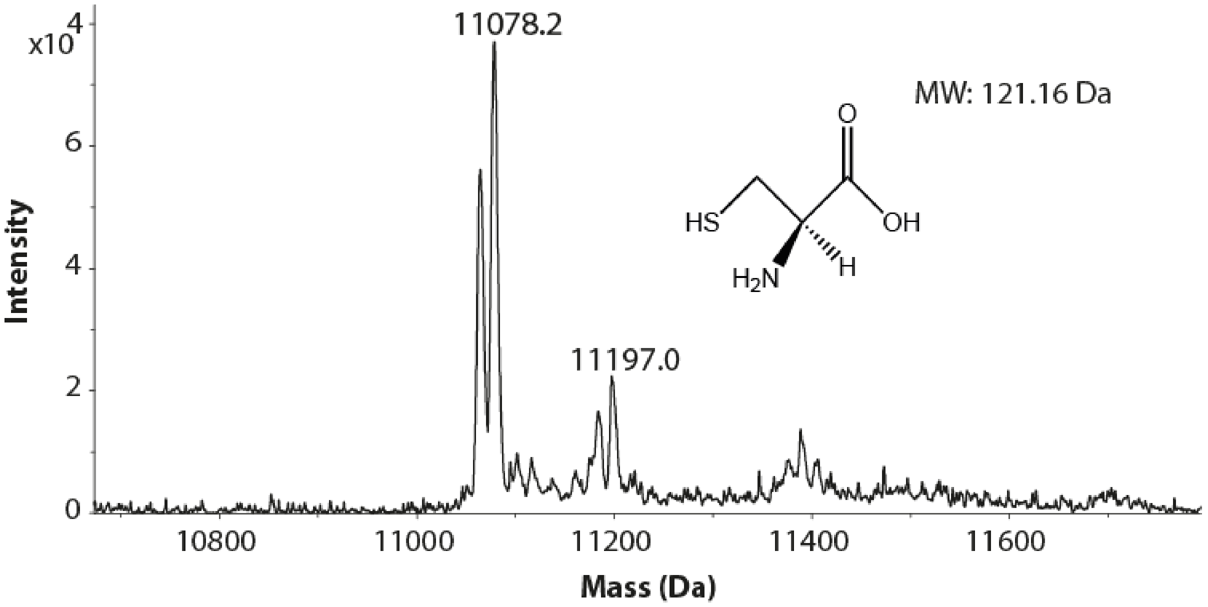

Supplementary Figure 15. Deconvoluted mass spectrum of I27$_{24/55}$ with the post-translational modification by l-cysteine. The deconvoluted mass spectrum of I27$_{24/55}$ in the presence of l-cysteine displays two peaks at a mass of 11184 Da and 11197 Da, corresponding to the modification of both the non-methylated and methylated protein at a single cysteine residue.

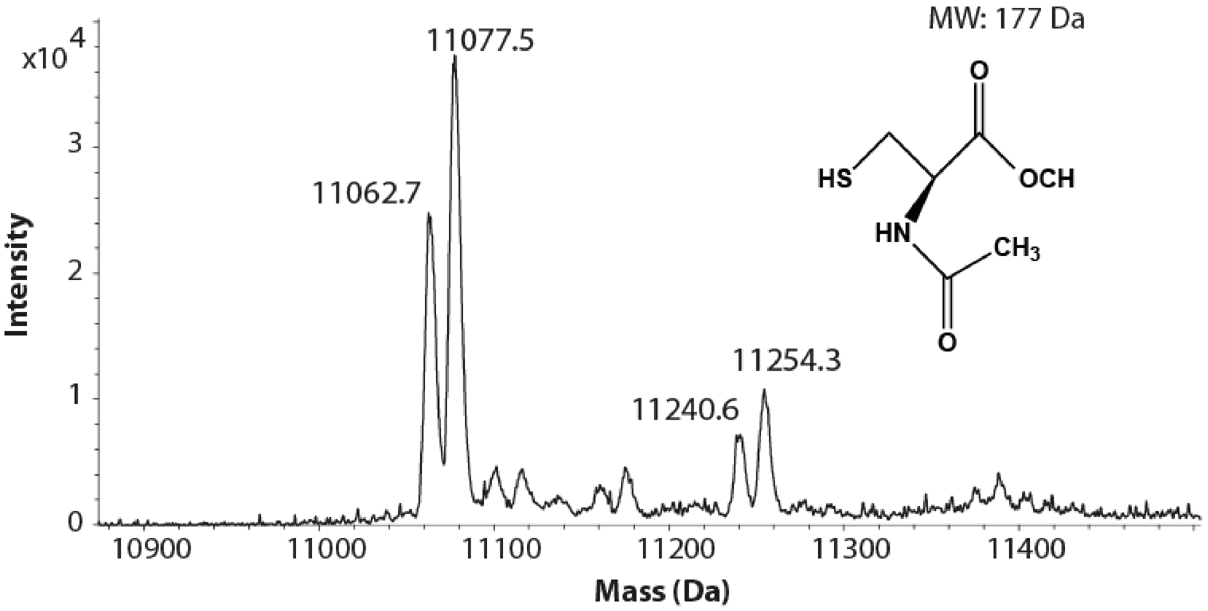

Supplementary Figure 16. Deconvoluted mass spectrum of I27$_{24/55}$ with the post-translational modification by *N*-acetyl-cysteine-methyl-ester. The deconvoluted mass spectrum of I27$_{24/55}$ in the presence of NAC-methyl-ester displays two peaks at a mass of 11240 Da and 11254 Da, corresponding to the modification of both the non-methylated and methylated protein at a single cysteine residue.

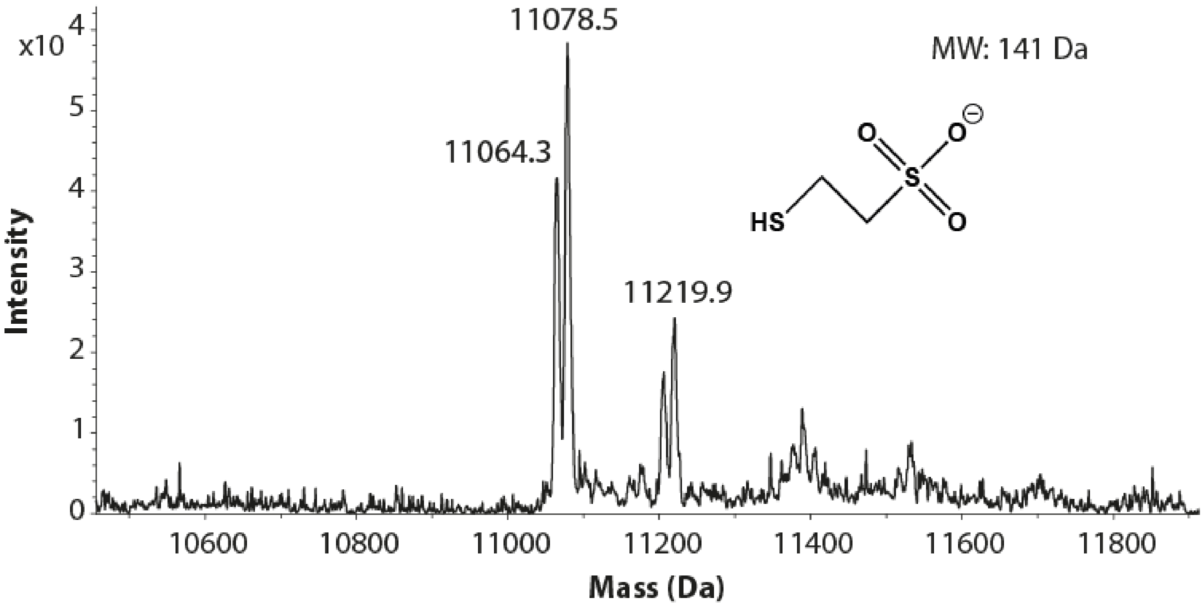

Supplementary Figure 17. Deconvoluted mass spectrum of I27$_{24/55}$ with the post-translational modification by mesna. The deconvoluted mass spectrum of I27$_{24/55}$ in the presence of mesna displays two peaks at a mass of 11206 Da and 11220 Da, corresponding to the modification of both the non-methylated and methylated protein at a single cysteine residue.

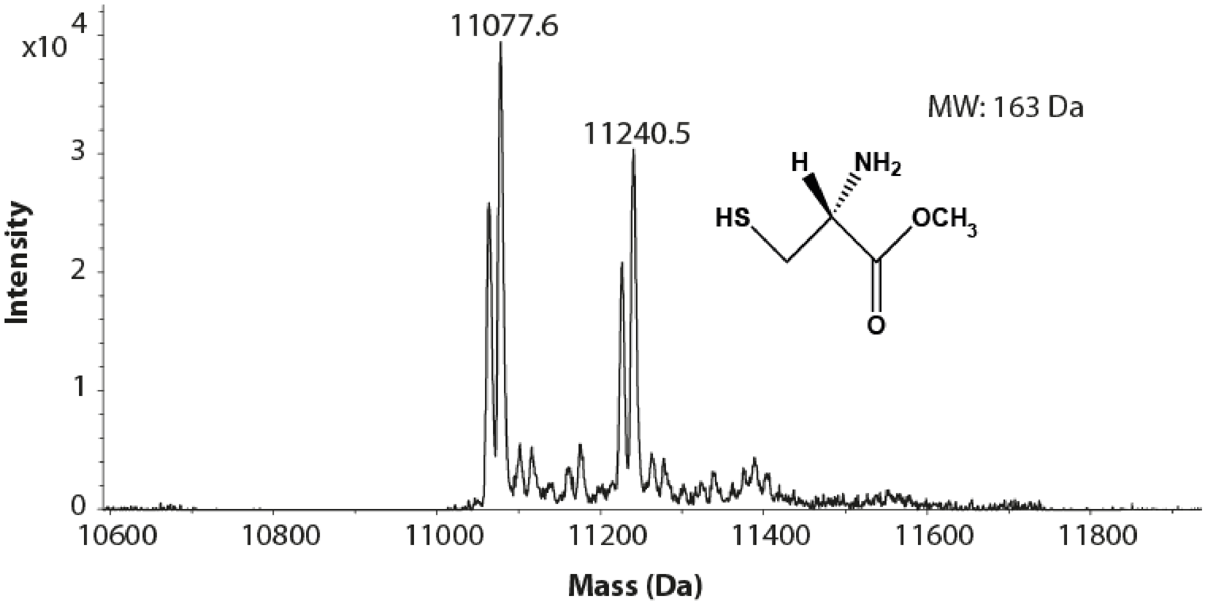

Supplementary Figure 18. Deconvoluted mass spectrum of $I27_{24/55}$ with the translational modification by *N*-acetyl-cysteine. The deconvoluted mass spectrum of $I27_{24/55}$ in the presence of NAC displays two peaks at a mass of 11226 Da and 11240 Da, corresponding to the modification of both the non-methylated and methylated protein at a single cysteine residue.

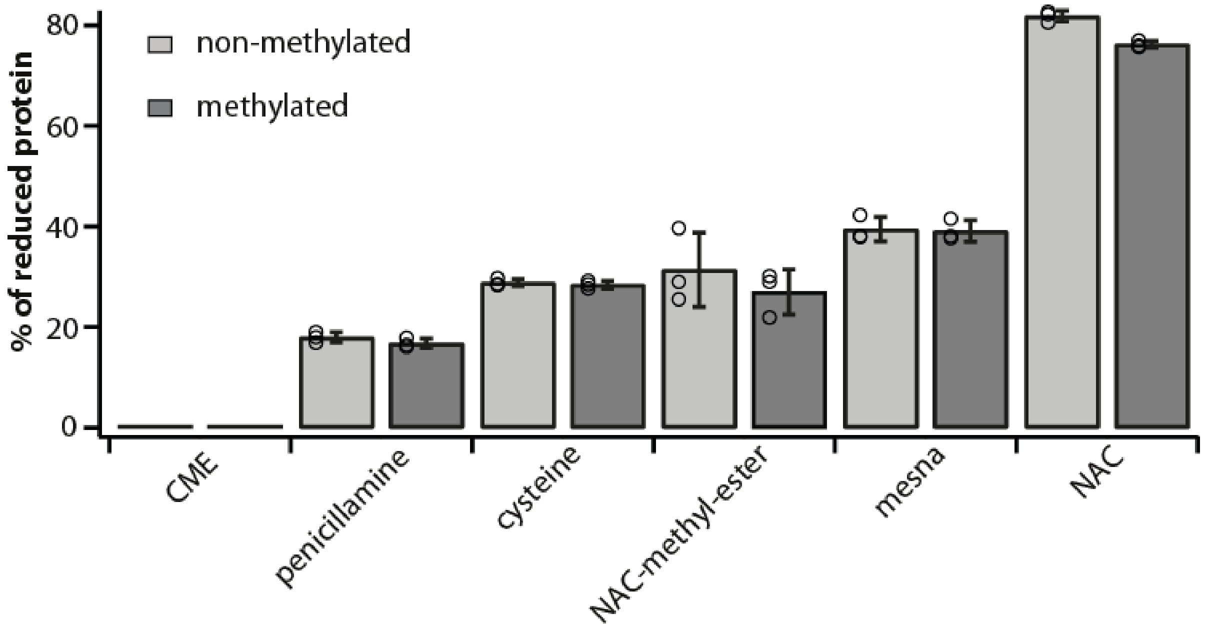

Supplementary Figure 19. The methylation of I27$_{24/55}$ does not alter the behaviour and reactivity of the protein. The methylation of I27$_{25/55}$ is present in the first 7 residues – very likely to be in the second Arg from the N-terminal, which is in the protein purification histidine tag– does not affect the percentage of disulfide bond reduction. Empty circles represent value from individual experiment (n=3 for each compound; error bars: s.d).

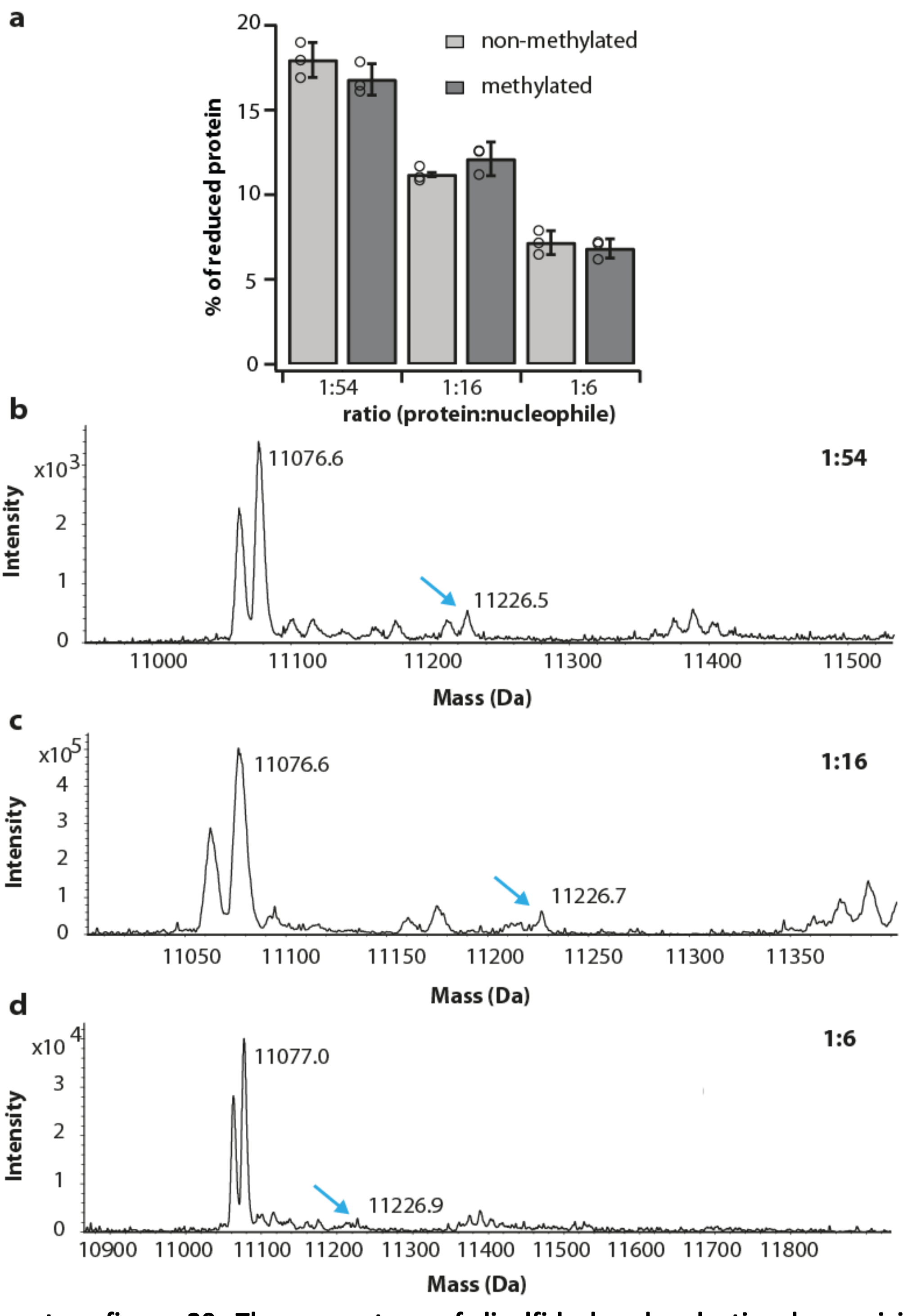

Supplementary figure 20. The percentage of disulfide bond reduction by penicillamine depends on the protein:nucleophile ratio. (a) The percentage of reduced $I27_{24/55}$ containing a single penicillamine modification depends on the concentration of deprotonated penicillamine. The percentage is obtained from the raw intensity, with no background subtraction, and therefore is likely to be a large overestimation of the actual percentage. The intensity of the 11266 Da peak (blue arrow) in the deconvoluted mass spectrum decreases with decreasing protein:penicillamine ratio. Empty circles represent value from individual experiment (n=3 for each condition; error bars: s.d) **(b)** 1:54 **(c)** 1:16, and **(d)** 1:6.

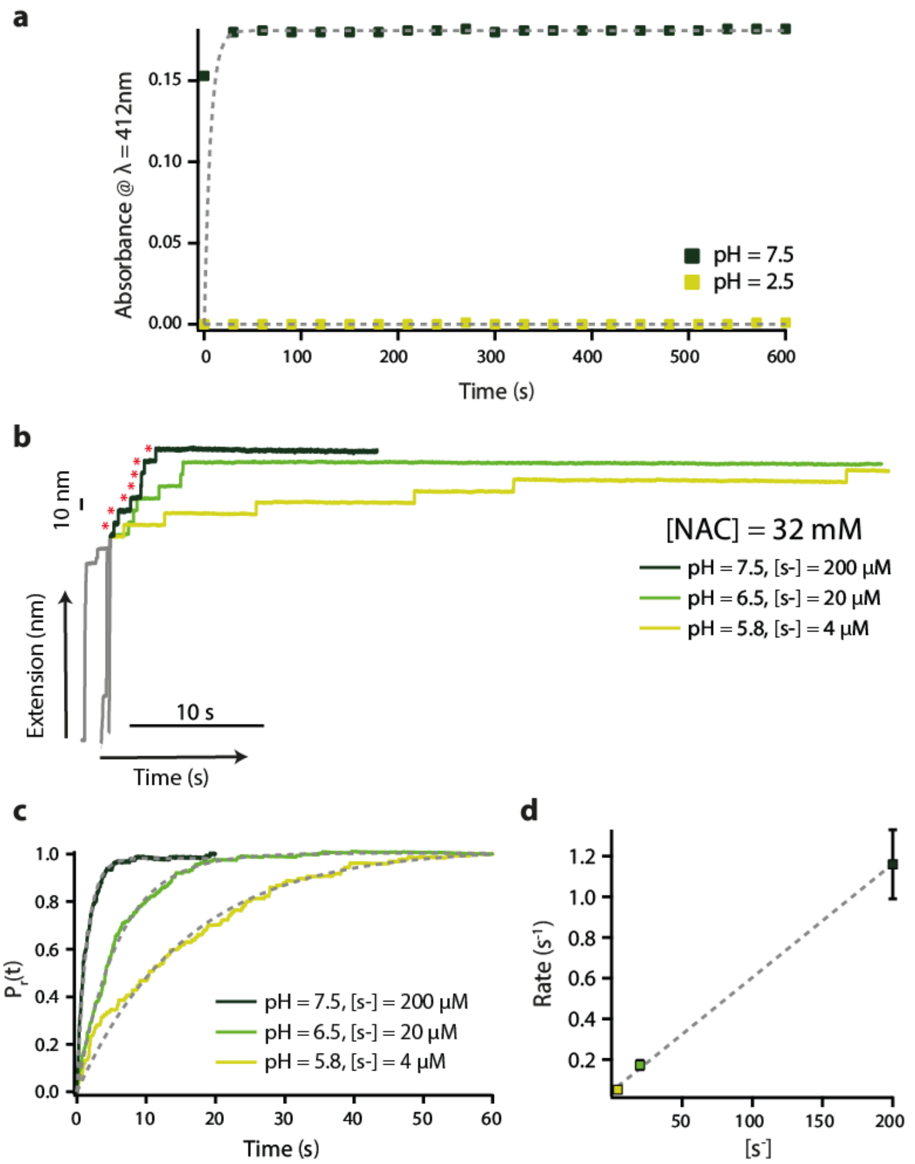

Supplementary figure 21. The kinetics of disulfide bond reduction is largely dominated by the concentration of deprotonated thiols. (**a**) We conducted the Ellman's assay at two pH values using the same total concentration (0.02 mM) of NAC, thereby modulating the concentration of the deprotonated species. At pH = 7.5 (dark green), approximately 0.62% of NAC is in the deprotonated form and the reduction of all DTNB molecules is almost instantaneous. By contrast, using the same assay performed at pH = 2.5, where 0.000006% of NAC molecules are deprotonated, we do not measure any change in absorbance over 10 mins. (**b**) A two-force protocol first unfolds the protein structure (grey) and then measures the kinetics of disulfide bond reduction in the presence of NAC at different pH values (green coloured). All trajectories are acquired at the same total concentration of NAC (32mM), however in each case the concentration of the deprotonated species is modulated by adjusting the pH. The fastest kinetics (dark green) corresponds to pH = 7.5, whereas the slowest (light green) corresponds to pH = 5.8. (**c**) The rate of disulfide bond reduction at 350 pN measured at 3 different pH values reveals that the kinetics of bond cleavage depends on the concentration of deprotonated thiol species. (**d**) The rate of disulfide bond reduction scales linearly with the concentration of the deprotonated species as expected for a first-order law that can be described as $r(F) = k(F)[S^-]$, (From left to right n= 14, 22 and 26; error bars: s.d).

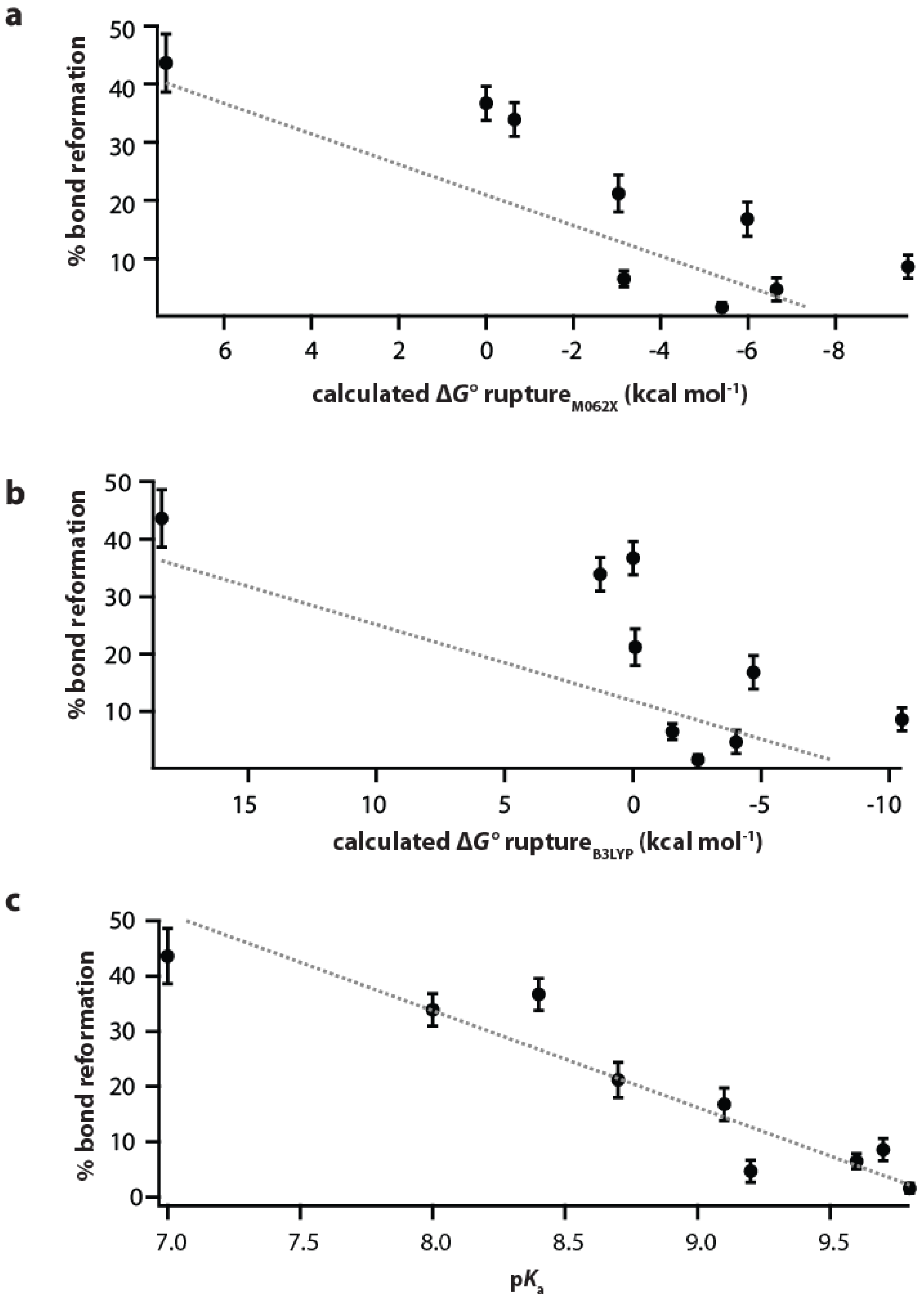

Supplementary Figure 22. Qualitative agreement between the percentage of disulfide reformation, the calculated Δ*G*° of rupture and the experimentally determined p*K*$_a$ values. **(a)** Using the M06-2X functional with one explicit water, **(b)** the B3LYP functional in purely implicit solvent **(c)** and the experimentally obtained p*K*$_a$ values (From left to right n=16, 35, 30, 24, 32, 33, 53, 31 and 39; all error bars: s.d).

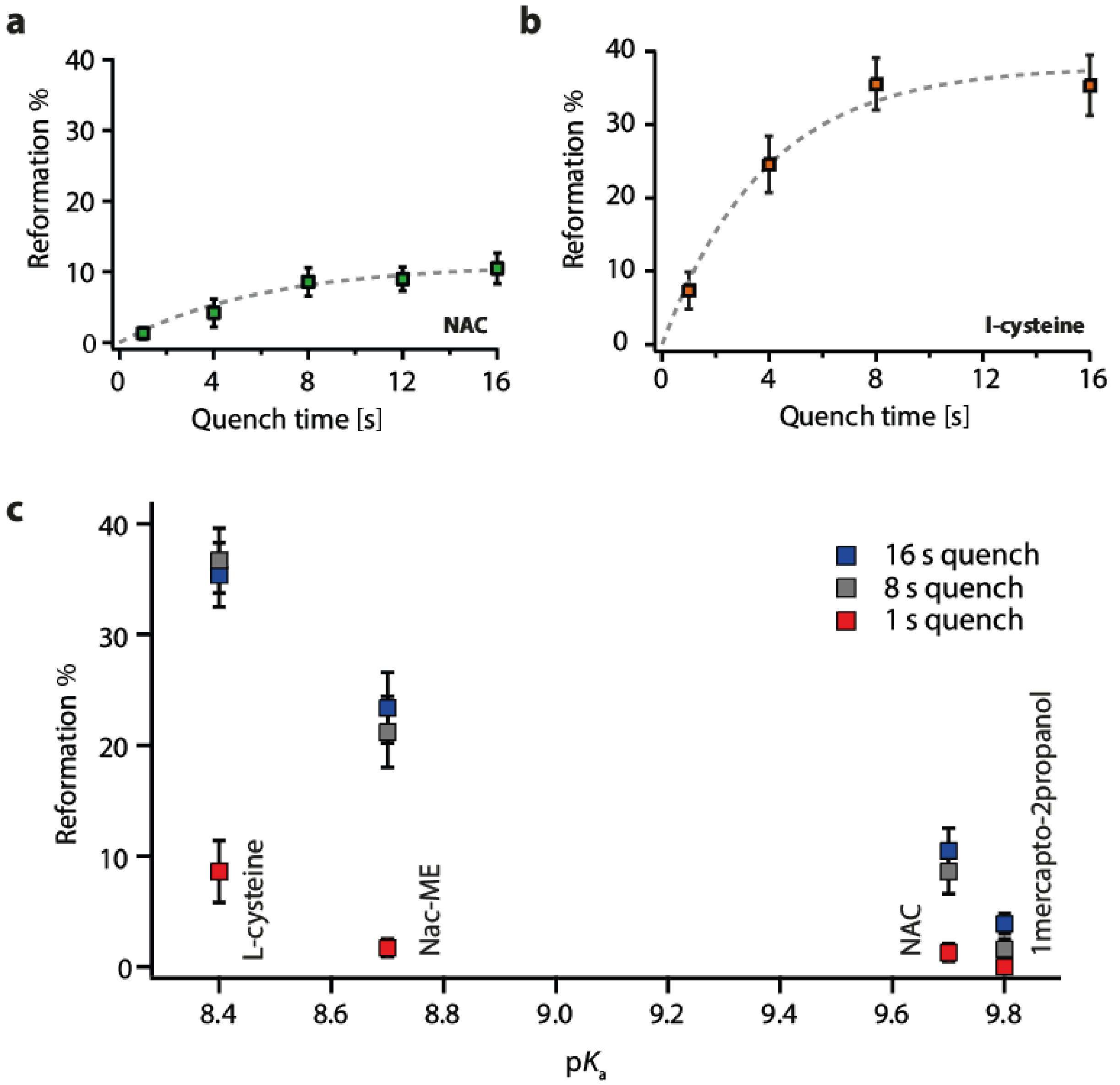

Supplementary Figure 23. Kinetics of disulfide bond reformation. (**a**) The percentage of disulfide bond reformation in the presence of NAC increases exponentially as a function of the time t_q spent at 0 pN, with an associated rate of reformation of k_{re} = 0.16 s^{-1} (grey dashed line) (From left to right n= 24, 20, 31, 36 and 38) . (**b**) Performing the same quench-time dependency experiments in the presence of l-cysteine provides qualitatively similar results, whereby the reformation efficiency exponentially increases with t_q, yielding k_{re} = 0.26 s^{-1} (From left to right n= 19, 22, 30 and 22). (**c**) Investigating the reformation percentage at t_q = 1s, t_q = 8s, and t_q = 16s for four different compounds, spanning a wide range of pK_a values (8.4 – 9.8), demonstrates that while a kinetic barrier is present, the t_q = 8s quench time used throughout the manuscript is enough to overcome the kinetic barrier, hence probing the thermodynamics of the reverse reaction (From 1s quench to 16s $n_{NAC\text{-}ME}$=34, 24 and 19; $n_{1mercapto\text{-}2propanol}$=19, 39 and 19; all error bars: s.d).

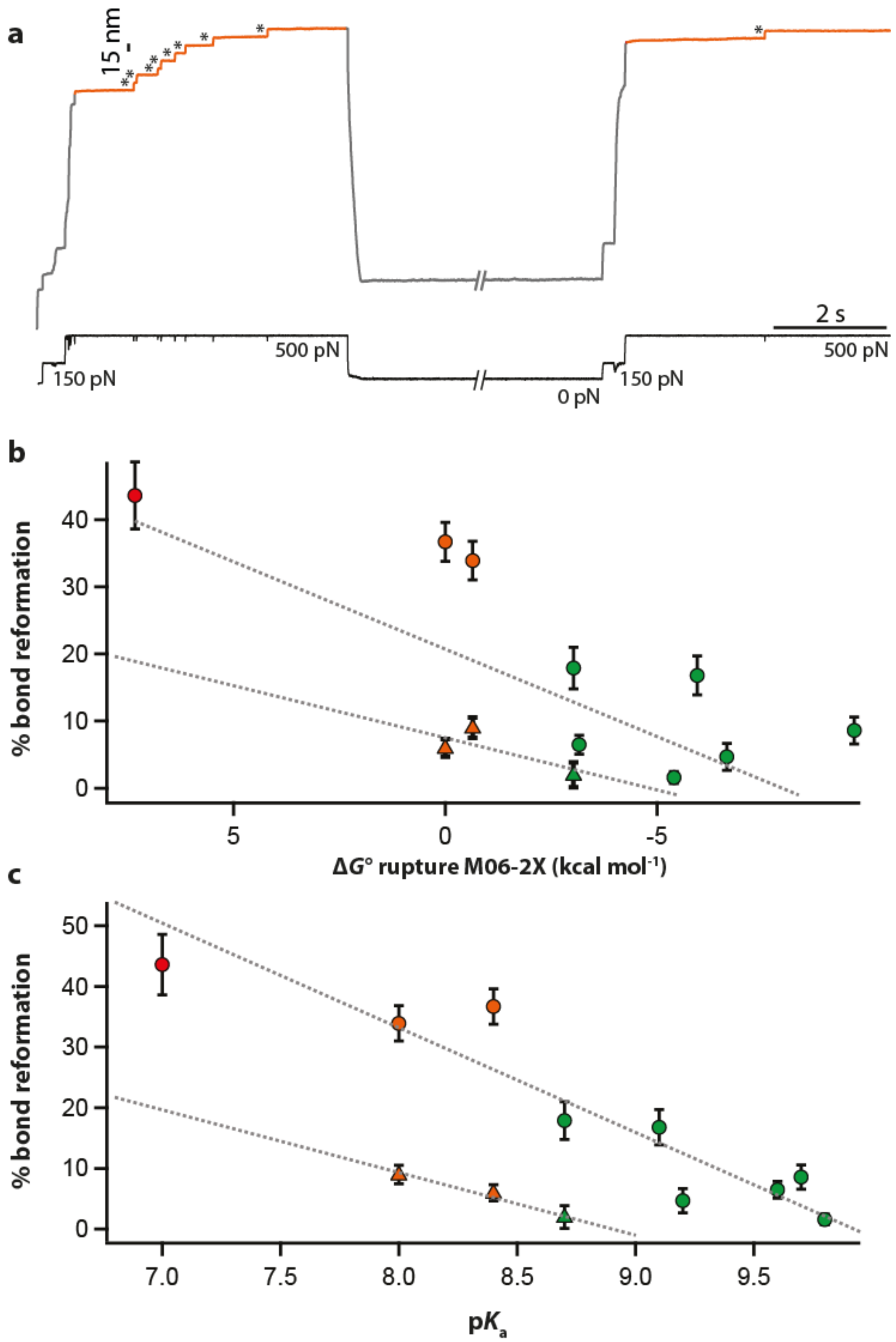

Supplementary Figure 24. The correlation between the energetics of the mechanochemical reaction and the reversibility is recapitulated in the homologous protein I27$_{32/75}$. (a) Disulfide bond rupture by penicillamine in the structurally homologous protein I27$_{32/75}$, which contains a single disulfide bond between residues 32 and 75, is hallmarked by steps of ~15 nm. **(b)** The relationship between the energetics of the disulfide rupture and the subsequent reversibility observed in I27$_{24/55}$ (circles) is observed in I27$_{32/75}$ (triangles), albeit with a lower disulfide yield. **(c)** The same trend is observed when considering the pK_a of the attacking thiol (From left to right bond reformation events of I27$_{32/75}$ n= 20, 20 and 13; all error bars: s.d).

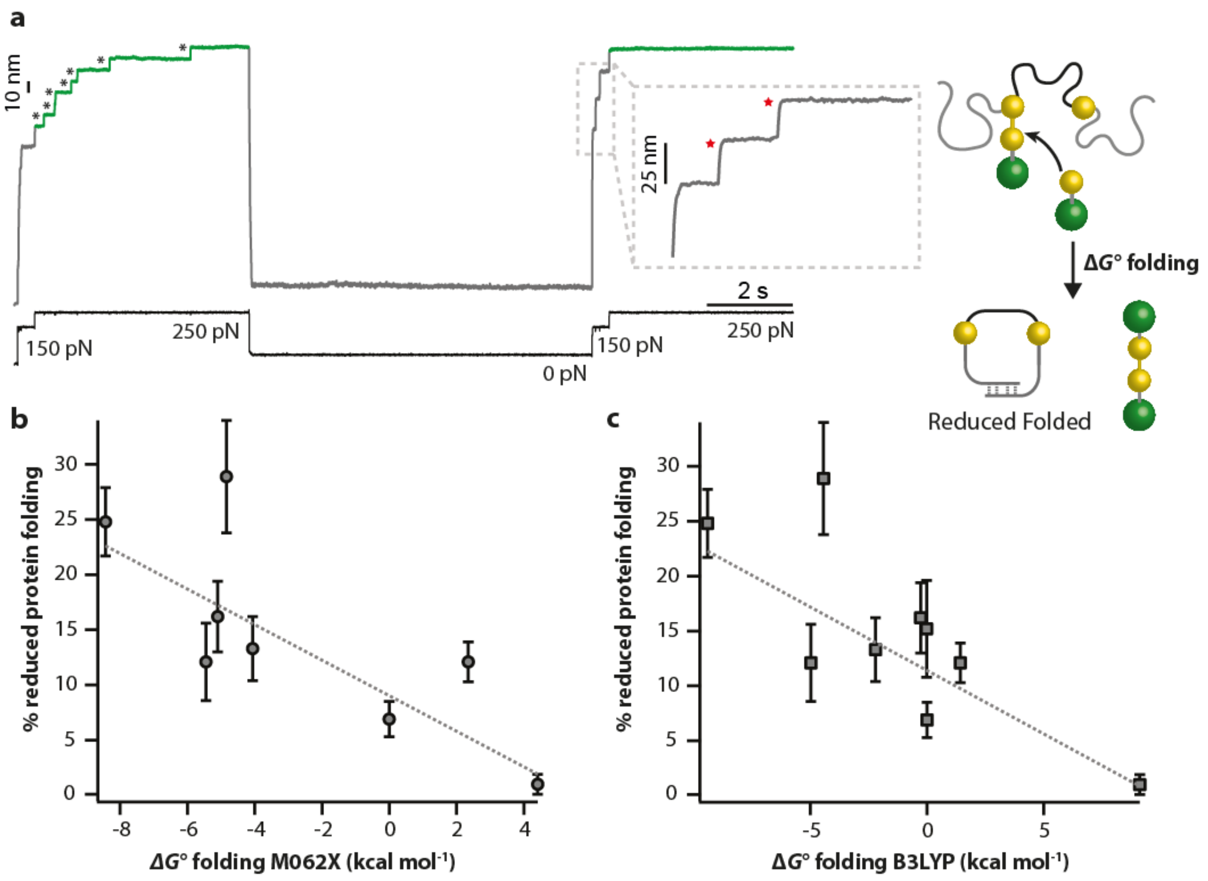

Supplementary Figure 25. The percentage of proteins that successfully refold in the absence of a disulfide bond is well predicted by DFT calculations. (a) The 5-pulse mechanochemical assay used in this study can readily distinguish whether a protein has refolded with or without a disulfide bond in the structure. While disulfide reformation is identified in the *test* pulse by steps of ~15 nm followed by ~10 nm, when a protein folds void of a disulfide is hallmarked by ~25 nm steps (*inset:* red asterisks). This scenario occurs when a free thiolate in solution attacks the mixed disulfide, removing the modification from the protein and forming a homodimer species. As such, the protein harbours two reduced cysteines and is able to effectively refold into its native structure, harbouring a mechanical stability that is intermediate between a misfolded conformation harbouring a mixed disulfide bond, and a stiff protein form that hallmarks a successfully refolded and oxidised protein. The occurrence of the attack of the mixed disulfide by solution thiolates can be rationalized by the results of DFT free energy calculations using either the **(b)** M062X, or **(c)** B3LYP functionals (from left to right n= 31, 18, 30, 38, 49, 21, 30, 31 and 16; all error bars: s.d).

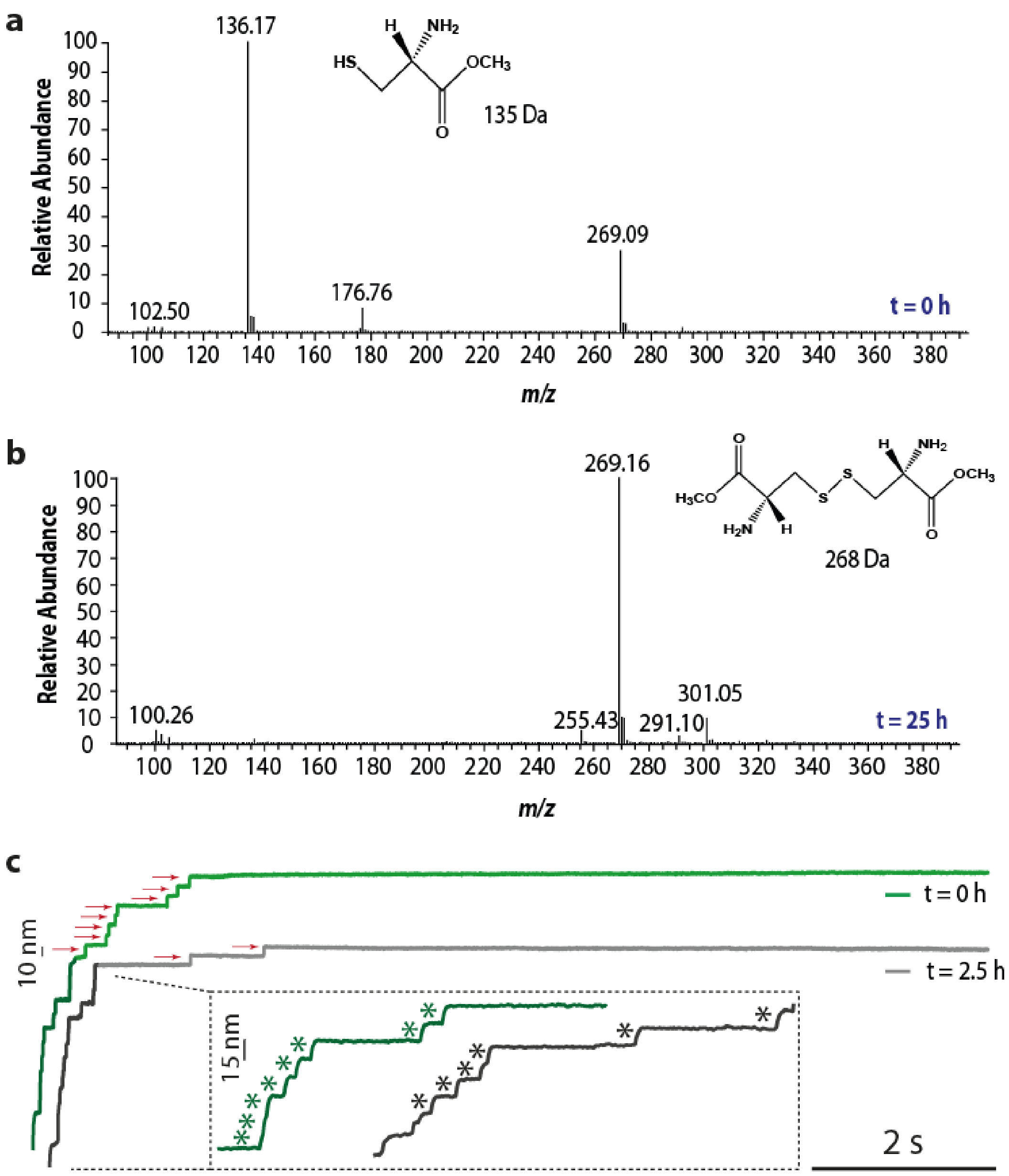

Supplementary Figure 26. Cysteine-methyl-ester displays time dependent dimerization. (a) Mass spectrometry measurements immediately after the preparation of cysteine-methyl-ester (0.2 mM) reveal that the majority of the compound is in monomeric form (*m/z* = 136 Da). **(b)** Mass spectrometry measurements taken 25 hours after solution preparation reveal that cysteine-methyl-ester has completely dimerized (*m/z* = 269 Da). **(c)** This dimerization is readily observed in the single molecule experiments. Disulfide rupture traces obtained immediately after cysteine-methyl-ester preparation (green), consistently display all 8 rupture events within the first 10 s (red arrows). However, traces obtained after 2.5 hours (grey), reveal that while 6 protein unfolding events are observed (*inset:* 15 nm steps, black asterisks), only 2 disulfide bond rupture events subsequently occur (red arrows). This dramatic slowing of the rate of disulfide cleavage signifies a reduction in the concentration of attacking species. For this reason, our single molecule exeriments were limited to ~3 hours.

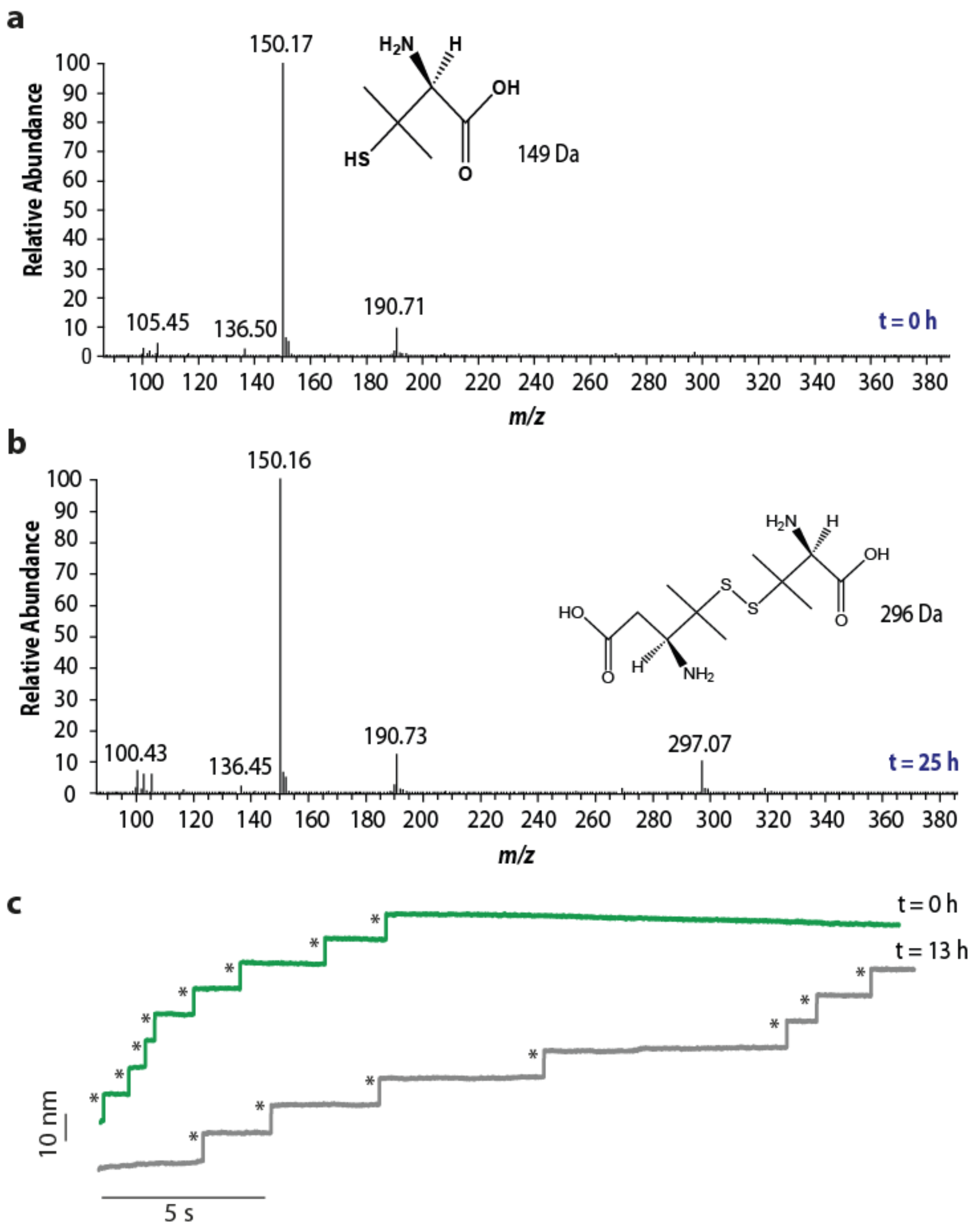

Supplementary Figure 27. Penicillamine displays time dependent dimerization. (a) Mass spectrometry measurements immediately after the preparation of penicillamine (0.2 mM) reveal that all of the compound is in monomeric form (*m/z* = 150 Da). **(b)** Mass spectrometry measurements taken 25 hours after solution preparation reveal that penicillamine has begun to dimerize (*m/z* = 297 Da). **(c)** This dimerization is readily observed in the single molecule experiments, where there is a dramatic reduction in the rate of disulfide cleavage between the start of the experiment (green) and after 13 hours (grey). For this reason, our single molecule exeriments were limited to ~3 hours.

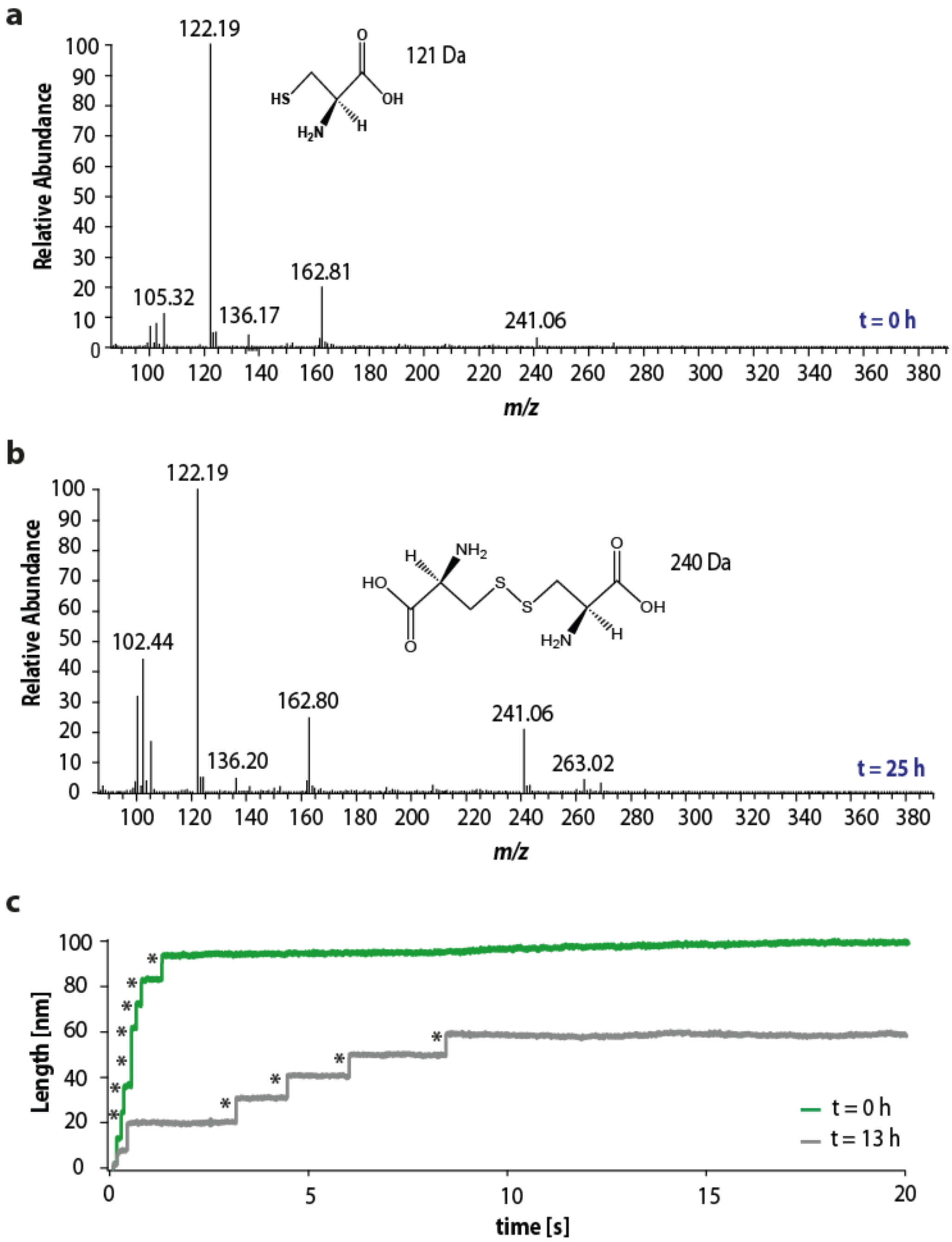

Supplementary Figure 28. L-cysteine displays time dependent dimerization. (a) Mass spectrometry measurements immediately after the preparation of L-cysteine (0.2mM) reveal that the majority of the compound is in monomeric form (*m/z* = 122 Da). **(b)** Mass spectrometry measurements taken 25 hours after solution preparation reveal that cysteine has begun to dimerize (*m/z* = 241 Da). **(c)** This dimerization is readily observed in the single molecule experiments. There is a dramatic reduction in the rate of disulfide cleavage between the start of the experiment (green) and after 13 hours (grey). For this reason, our single molecule exeriments were limited to ~3 hours.

Supplementary Tables

Supplementary Table 1. A comparison of the DFT values obtained for each compound using the M062X and B3LYP functionals.

**Compound**	**Experimental p*k*$_a$**	**p*k*$_a$ M062X**	**Δ*G*° rupture M062X (kcal mol^{-1})**	**Charge M062X thiolate (S^-) (e)**	**Charge M062X thiol (SH) (e)**
Cys-ME	7.0	5.15	7.33	-0.68416	-0.04662
Penicillamine	8.0	6.93	-0.65	-0.65645	-0.11281
Cysteine	8.4	7.57	0.00	-0.69014	-0.07931
NAC-ME	8.7	10.05	-3.03	-0.70013	-0.08063
glycerol	9.6	10.80	-3.16	-0.7139	-0.07477
1-mercapto	9.8	11.10	-5.40	-0.72031	-0.12777
Glutathione	9.1	13.43	-5.98	-0.70405	-0.11289
Mesna	9.2	11.35	-6.65	-0.72402	-0.09777
NAC	9.7	12.83	-9.66	-0.72283	-0.16438
	**Experimental p*k*$_a$**	**p*k*$_a$ B3LYP**	**Δ*G*° rupture B3LYP (kcal mol^{-1})**	**Charge B3LYP thiolate (S^-) (e)**	**Charge B3LYP thiol (SH) (e)**
Cys-ME	7.0	4.75	18.37	-0.66635	0.01076
Penicillamine	8.0	9.06	1.28	-0.70348	-0.07013
Cysteine	8.4	8.96	0.00	-0.73155	-0.04734
NAC-ME	8.7	12.64	-0.08	-0.75114	-0.03988
Thioglycerol	9.6	12.30	-1.53	-0.77940	-0.04171
1-mercapto	9.8	12.84	-2.53	-0.79299	-0.10336
Glutathione	9.1	12.05	-4.69	-0.77961	-0.06073
Mesna	9.2	14.83	-4.02	-0.80730	-0.07477
NAC	9.7	15.84	-10.48	-0.80838	-0.12509

Supplementary Table 2. Free-energy landscape parameters obtained from the single molecule force dependency data.

**Compound**	**Δx (Å)**	***k*(0) (M^{-1} s^{-1})**	**Δ*G*$^{\neq}$ (kT)**
Glutathione	0.34 ± 0.03	86.5	11.7
Mesna	0.33 ± 0.04	131.4	11.2
Thioglycerol	0.37 ± 0.02	134.3	11.2
NAC	0.37 ± 0.21	244.1	10.6

Supplementary Table 3. Sequences of all protein constructs used in the manuscript. The six histidine residues at the beginning are used for purification. The polyprotein constructs for single molecule experiments have a cysteine tag at the end for attachment to the gold coverslides.

Protein Construct	Polyprotein Sequence
$(I27_{E24C\text{-}K55C})_8$	MRGSHHHHHHGSLIEVEKPLYGVEVFVGETAHFEICLSEPDVHGQWKLKG<br>QPLAASPDAEIIEDGKCHILILHNAQLGMTGEVSFQAANTKSAANLKVKELR<br>SLIEVEKPLYGVEVFVGETAHFEICLSEPDVHGQWKLKGQPLAASPDAEIIED<br>GKCHILILHNAQLGMTGEVSFQAANTKSAANLKVKELRSLIEVEKPLYGVEV<br>FVGETAHFEICLSEPDVHGQWKLKGQPLAASPDAEIIEDGKCHILILHNAQL<br>GMTGEVSFQAANTKSAANLKVKELRSLIEVEKPLYGVEVFVGETAHFEICLSE<br>PDVHGQWKLKGQPLAASPDAEIIEDGKCHILILHNAQLGMTGEVSFQAAN<br>TKSAANLKVKELRSLIEVEKPLYGVEVFVGETAHFEICLSEPDVHGQWKLKG<br>QPLAASPDAEIIEDGKCHILILHNAQLGMTGEVSFQAANTKSAANLKVKELR<br>SLIEVEKPLYGVEVFVGETAHFEICLSEPDVHGQWKLKGQPLAASPDAEIIED<br>GKCHILILHNAQLGMTGEVSFQAANTKSAANLKVKELRSLIEVEKPLYGVEV<br>FVGETAHFEICLSEPDVHGQWKLKGQPLAASPDAEIIEDGKCHILILHNAQL<br>GMTGEVSFQAANTKSAANLKVKELRSLIEVEKPLYGVEVFVGETAHFEICLSE<br>PDVHGQWKLKGQPLAASPDAEIIEDGKCHILILHNAQLGMTGEVSFQAAN<br>TKSAANLKVKELRSRSCC
$(I27_{G32C\text{-}A75C})_8$	MRGSHHHHHHGSLIEVEKPLYGVEVFVGETAHFEIELSEPDVHCQWKLKG<br>QPLAASPDAEIIEDGKKHILILHNAQLGMTGEVSFQCANTKSAANLKVKELR<br>SLIEVEKPLYGVEVFVGETAHFEIELSEPDVHCQWKLKGQPLAASPDAEIIED<br>GKKHILILHNAQLGMTGEVSFQCANTKSAANLKVKELRSLIEVEKPLYGVEV<br>FVGETAHFEIELSEPDVHCQWKLKGQPLAASPDAEIIEDGKKHILILHNAQL<br>GMTGEVSFQCANTKSAANLKVKELRSLIEVEKPLYGVEVFVGETAHFEIELSE<br>PDVHCQWKLKGQPLAASPDAEIIEDGKKHILILHNAQLGMTGEVSFQCANT<br>KSAANLKVKELRSLIEVEKPLYGVEVFVGETAHFEIELSEPDVHCQWKLKGQ<br>PLAASPDAEIIEDGKKHILILHNAQLGMTGEVSFQCANTKSAANLKVKELRSL<br>IEVEKPLYGVEVFVGETAHFEIELSEPDVHCQWKLKGQPLAASPDAEIIEDGK<br>KHILILHNAQLGMTGEVSFQCANTKSAANLKVKELRSLIEVEKPLYGVEVFV<br>GETAHFEIELSEPDVHCQWKLKGQPLAASPDAEIIEDGKKHILILHNAQLGM<br>TGEVSFQCANTKSAANLKVKELRSLIEVEKPLYGVEVFVGETAHFEIELSEPD<br>VHCQWKLKGQPLAASPDAEIIEDGKKHILILHNAQLGMTGEVSFQCANTKS<br>AANLKVKELRSCC
$I27_{E24C\text{-}K55C}$	MRGSHHHHHHGSLIEVEKPLYGVEVFVGETAHFEICLSEPDVHGQWKLKG<br>QPLAASPDAEIIEDGKCHILILHNAQLGMTGEVSFQAANTKSAANLKVKEL

Supplementary Table 4. Molecular fragmentation peaks produced by Collision Induced Dissociation (CID) of the non-methylated $I27_{24/55}$ monomer (11062.5 Da).

Name	Ion Type	Ion Number	Theoretical Mass	Observed Mass	Mass Difference (Da)	Mass Difference (ppm)
B7	B	7	856.39	856.38	-0.010	-11.83
B8	B	8	993.45	993.44	-0.011	-11.32
B9	B	9	1130.51	1130.49	-0.013	-11.41
B10	B	10	1267.56	1267.55	-0.015	-11.44
B14	B	14	1637.79	1637.77	0.019	-11.43
B15	B	15	1766.83	1766.81	0.020	-11.11

Supplementary Table 5. Molecular fragmentation peaks produced by Collision Induced Dissociation (CID) of the methylated $I27_{24/55}$ monomer (+14 Da peak, 11076.5 Da).

Name	Ion Type	Ion Number	Theoretical Mass	Observed Mass	Mass Difference (Da)	Mass Difference (ppm)
B6	B	6	705.31	705.30	-0.009	-12.60
B7	B	7	842.37	842.36	-0.010	-12.26
B8	B	8	979.43	979.42	-0.013	-13.49
B14	B	14	1623.77	1623.75	-0.020	12.59
B15	B	15	1752.81	1752.79	-0.023	12.87
B16	B	16	1851.88	1851.86	-0.023	-12.84

Supplementary Methods

Mass spectrometry of Cys-ME, Penicillamine and L-cysteine

The formation of dimers was determined by ESI-MS experiments, performed with a Thermo LTQ XL linear trap mass spectrometer with electron transfer dissociation source. The electrospray voltage was kept at 4.00 kV, the capillary temperature was set at 350 °C and the capillary voltage was 19V. A full MS (*m/z* 86-700 Da) scan was acquired right after sample preparation and again after 25 h, with a nominal mass resolution. Samples were delivered by means of automatic flow injection. MS data was analysed with Xcalibur version 2.2. All three monomers were confirmed by following the *m/z* positive molecular ion peak at each corresponding molecular mass. Dimers of each compound were confirmed by following the *m/z* positive molecular ion after acquiring the spectrums 25 h after sample preparation. MS experiments were performed at pH of 7.5 and a final concentration of 200 µM in a 50 mM ammonium bicarbonate buffer (Sigma-Aldrich, 99.5%).

Top down MS/MS identification of protein methylation

Top down electrospray CID MS/MS spectra were acquired on a LQT-Orbitrap Velos Pro. Raw files were deconvoluted using Xcalibur/Xtract and fragment ions mapped using Prosight Lite software. This enabled localisation of a +14 Da modification to the first seven amino acids of the N-terminus. The most likely explanation is arginine methylation. Prosight analysis was used to confirm the presence of the oxidized form of $I27_{24/55}$.
